# Zinc enhances the cellular energy supply to improve cell motility and restore impaired energetic metabolism in a toxic environment induced by OTA

**DOI:** 10.1038/s41598-017-14868-x

**Published:** 2017-11-07

**Authors:** Xuan Yang, Haomiao Wang, Chuchu Huang, Xiaoyun He, Wentao Xu, Yunbo Luo, Kunlun Huang

**Affiliations:** 1 Beijing Advanced Innovation Center for Food Nutrition and Human Health, College of Food Science & Nutritional Engineering, China Agricultural University, Beijing, 100083 China; 2 Beijing Laboratory for Food Quality and Safety, Beijing, 100083 China

## Abstract

Exogenous nutrient elements modulate the energetic metabolism responses that are prerequisites for cellular homeostasis and metabolic physiology. Although zinc is important in oxidative stress and cytoprotection processes, its role in the regulation of energetic metabolism remains largely unknown. In this study, we found that zinc stimulated aspect in cell motility and was essential in restoring the Ochratoxin A (OTA)-induced energetic metabolism damage in HEK293 cells. Moreover, using zinc supplementation and zinc deficiency models, we observed that zinc is conducive to mitochondrial pyruvate transport, oxidative phosphorylation, carbohydrate metabolism, lipid metabolism and ultimate energy metabolism in both normal and toxic-induced oxidative stress conditions *in vitro*, and it plays an important role in restoring impaired energetic metabolism. This zinc-mediated energetic metabolism regulation could also be helpful for DNA maintenance, cytoprotection and hereditary cancer traceability. Therefore, zinc can widely adjust energetic metabolism and is essential in restoring the impaired energetic metabolism of cellular physiology.

## Introduction

Emerging evidence from animal models has indicated a fundamental interplay between micronutrients and metabolic physiology with implications for health and disease^1,2^. An impaired energetic metabolism is often accompanied by a shortage of cellular energy supply and vice versa. Nutrient availability and stress stimuli are important factors that regulate cellular energy levels in response to pivotal changes in cellular homeostasis. The disruption of this balance can potentially lead to a number of pathologies, including diabetes, cancer and cardiovascular disease^3^. Cell motility is a fundamental phenomenon and has been implicated in a variety of biological processes, from morphogenesis, metastasis and the immune response to the development of pathologies such as cancer growth^4–7^. The cell motility of mechanical property changes also has an impact on many important biological behaviors of cells, including adhesion, division, differentiation and deformation. Controlled energy metabolism makes all of these behaviors feasible^8^.

Cells have evolved a highly integrated network of mechanisms to utilize micronutrient and to coordinate cellular energy metabolism, survival/death, proliferation, differentiation and repair with metabolic states^9^. Zinc is an essential trace metal and acts as a cofactor for numerous enzymes and transcription factors that require it for various cellular functions^10,11^. It has been known for decades that zinc plays a key role in the regulation of the cellular secretory phenotype, cell cycle and apoptosis^12–14^. Dietary consumption of zinc in humans is related to the levels of plasma glucose, tumor necrosis factors (TNFs), interleukins (ILs), zinc transporters (ZnTs) and metallothioneins (MTs), such zinc metabolism is related to certain disorders such as metabolic syndrome, diabetic complications and immunodeficiency syndrome^11,15,16^. Zinc deficiency sensitizes cells to oxidative stress^17^. A number of articles have reported that appropriate zinc supplementation can attenuate toxin-induced toxicity and diseases^14,18–20^, however, the intake of an inappropriate concentration of zinc is considered a risk factor for neurotoxicity and the endoplasmic reticulum stress response^21^. Ochratoxin A (OTA) has definite cytotoxicity and can cause mitochondrial damage and energy metabolism disturbance by increasing the mitochondrial membrane potential, disrupting the mitochondrial electron transport chain and perturbing the TCA cycle^22–24^, thereby, inhibiting the generation of ATP^25^. Moreover, because of impaired energy metabolism, OTA can also lead to disorders of glucose metabolism, lipid metabolism and cell senescence in *vivo* and *vitro* ^26–28^.

The bulk of cellular energy is mainly generated in the mitochondria, in the form of ATP through oxidative phosphorylation (OXPHOS) and participation in essential cellular processes^29,30^. Lon peptidase 1 (Lonp1) is a mitochondrial DNA binding protein that is important for mtDNA maintenance. It is associated with the tricarboxylic acid cycle and plays a key role in metabolic reprogramming by remodeling OXPHOS complexes^31,32^. In eukaryotic cells, pyruvate is one of the most important metabolic factors; its uptake can be controlled by the mitochondrial pyruvate carrier (MPC), and after uptake, it cannot be oxidized for efficient ATP production^33^. Mitochondrial biogenesis and glucose and fatty acid metabolism are regulated by peroxisome proliferator-activated receptor γ coactivator 1-a (PGC1α)^34^. PGC1α acts as a protective molecule against ROS generation and damage^35^. The peroxisome proliferator-activated receptor γ (PPARγ) is involved in the regulation of lipid and carbohydrate metabolism^36^. AMP-activated protein kinase (AMPK) represents an energy sensor and metabolic regulator and responds to imbalances in metabolic homeostasis and nutrition status *in vivo* ^37^. The important role of AMPK in the control of food intake is involved with acetyl-CoA carboxylase (ACC), a substrate of AMPK^38^. The enzymatic step following mitochondrial entry is the conversion of pyruvate to ACC by the pyruvate dehydrogenase (PDH) complex, in which the phosphorylation of PDH is specifically regulated by pyruvate dehydrogenase kinase (PDK), which controls the oxidative decarboxylation of pyruvate and the homeostasis of carbohydrate fuels in mammals. Moreover, PDK activity has been shown to decrease in individuals consuming a diet^39^. However, the function of zinc in the cellular energy supply or restoring impaired energetic metabolism is largely unknown. Except for PPARγ, the relationship between zinc and its regulators remains unclear. This study began by investigating the function of zinc in stimulated cell motility and motility speed in normal cell lines. Afterwards, energy metabolism pathways and impaired energetic metabolism models were introduced to show the function of zinc in the cellular energy supply.

## Materials and Methods

### Cell culture and treatment

Human embryonic kidney 293 cells (HEK293) were grown in Dulbecco’s modified Eagle’s medium (DMEM) supplemented with 10% FBS (HyClone, Utah, USA), 100 U/mL penicillin, 100 μg/mL streptomycin, 250 ng/mL amphotericin B (Macgene, Beijing, China) at 37 °C in 5% CO_2_ and 95% humidity. By the day of the experiment, the cells had usually proliferated to 70–80% confluence. Cells were incubated with 50 μM ZnSO_4_ (Sigma, Darmstadt, Germany), 5 μM TPEN, 25 μM OTA, 50 μM ZnSO_4_ and 25 μM OTA containing medium for 24 h. The images were captured with a BX-51 fluorescence microscope (Olympus, Tokyo, Japan). The cells were seeded on plates or in single wells and were dispersed with a solution of 0.25% trypsin (w/v) and 0.52 mM EDTA after the indicated exposure time (Macgene, Beijing, China).

### Cell viability

The cell viability was determined using Cell Counting Kit-8 (CCK-8, Beyotime, Beijing, China) according to the manufacturer’s instructions. Briefly, cells were seeded in a 96-well plate at a density of 1 × 10^4^ cells/well and were washed once with phosphate-buffered saline (PBS). Subsequently, 10 μL of WST-8 dye and 100 μL of PBS were added to each well. The cells were then incubated at 37 °C for 1 h. The absorbance at 450 nm was determined using a microplate reader (Thermo, Massachusetts, USA).

### Phase Holographic Imaging

Cells were seeded in a 6-well plate at 37 °C in 5% CO_2_, 95% humidity and shielded from light. The HoloMonitor^TM^ M3 digital holographic microscope (Phase Holographic Imaging AB, Lund, Sweden) records 3D information of cells using the hologram × 20 lens, and interfering wave fronts were induced by the exposure to a 0.8 mW HeNe laser (633 nm). The hologram can be displayed showing either the phase or amplitude information of the light wave. The motility refers to how far the cells have moved from their starting point at the beginning of the analysis until the current time point. The motility speed is the total cell movement per hour. The migration is the shortest distance between the starting point of the cell analysis and the current point. Image were analyzed in the HoloStudio^TM^ software, according to the instrument manual.

### ATP concentration detection

Cells were seeded in a 6-well plate at 37 °C in 5% CO_2_, 95% humidity and shielded from light. After treatment, the ATP Assay Kit (Beyotime, Beijing, China) and GloMax® 96 Microplate Luminometer (Promega, Wisconsin, USA) were used in the ATP concentration detection.

### SOD activity assay

The cells were lysed with 300 μL lysis buffer on ice and centrifuged at 12,000 rpm and 4 °C for 10 min. The supernatants were used to measure the SOD activity using an SOD kit (Jiancheng Bioengineering Institute, Namjing, China) according to the manufacturer’s protocol.

### Protein extraction and measurement

After harvest and washing with ice-cold PBS, cells were dissolved in RIPA buffer PMSF (150 mM sodium chloride, 1.0% Triton X-100, 0.5% sodium deoxycholate, 0.1% SDS, 50 mM Tris, protease and phosphatase inhibitor mixture (Beyotime, Beijing, China)). The cells were transferred to a 1 mL syringe and were then homogenized. The resulting cellular lysates were centrifuged at 14,000 rpm for 10 min at 4 °C. The supernatant proteins were collected and quantified using the BCA Protein Assay Kit (Beyotime, Beijing, China).

### Western blot analysis

Extracted protein from each sample were loaded onto 12.5% Tricine-SDS-PAGE gels, transferred to a PVDF membrane (Millipore, Massachusetts, USA), blocked in 5% (wt/vol) skim milk in TBST (0.02 M Tris base, 0.14 M NaCl, 0.1% Tween 20, pH 7.4), and incubated with primary antibodies overnight at 4 °C before being incubated with secondary antibodies conjugated with HRP. The following primary antibodies were used: rabbit Acetyl-CoA Carboxylase Antibody (#3662, CST, Massachusetts, USA, 1:1000), rabbit AMPKα Antibody (#2532, CST, USA, 1:2000), rabbit phosphoinositide-dependent kinase 1 (PDK1) Antibody (#3062, CST, USA, 1:1000), rabbit MPC1 Antibody (#14462, CST, USA, 1:2000), rabbit MPC2 Antibody (#46141, CST, USA, 1:1000), rabbit Phospho-Acetyl-CoA Carboxylase (Ser79) Antibody (#3661, CST, USA, 1:1000), rabbit Phospho-AMPKα (Thr172) Antibody (#2535, CST, USA, 1:1000), rabbit Lonp1 Antibody (15440, Proteintech, USA, 1:1000), rabbit PGC1α Antibody (ab54481, abcam, Cambridge, USA, 1:1000), rabbit PPARγ Antibody (#2443, CST, USA, 1:1000), mouse total OXPHOS Rodent WB Antibody Cocktail (ab110413, abcam, USA, 1:1000), and rabbit β-Actin Antibody (aa128, Beyotime, China, 1:1000). The restore PLUS Western Blot Stripping Buffer (Thermo, Massachusetts, USA) and the Super Signal West Pico chemiluminescent substrate (SageCreation, Beijing, China) were used for coloration of proteins. The total gray values of each band were digitized using BandScan V4.3. The relative expression level of each protein was normalized to a reference protein, and the resulting ratios in the control group were normalized to 1.

### Statistical analysis

All data are shown as averages ± standard deviation of the three single experiments. In the phase holographic imaging assays, at least 12 cells per treatment were used for the calculation. The data from the different treatments were subjected to a one-way analysis of variance (ANOVA), and the means were compared using Duncan’s multiple range test. The differences were considered significant for p-values less than 0.05.

## Results

### Zinc improved the motion performance of HEK293 cells

**Figure 1:**
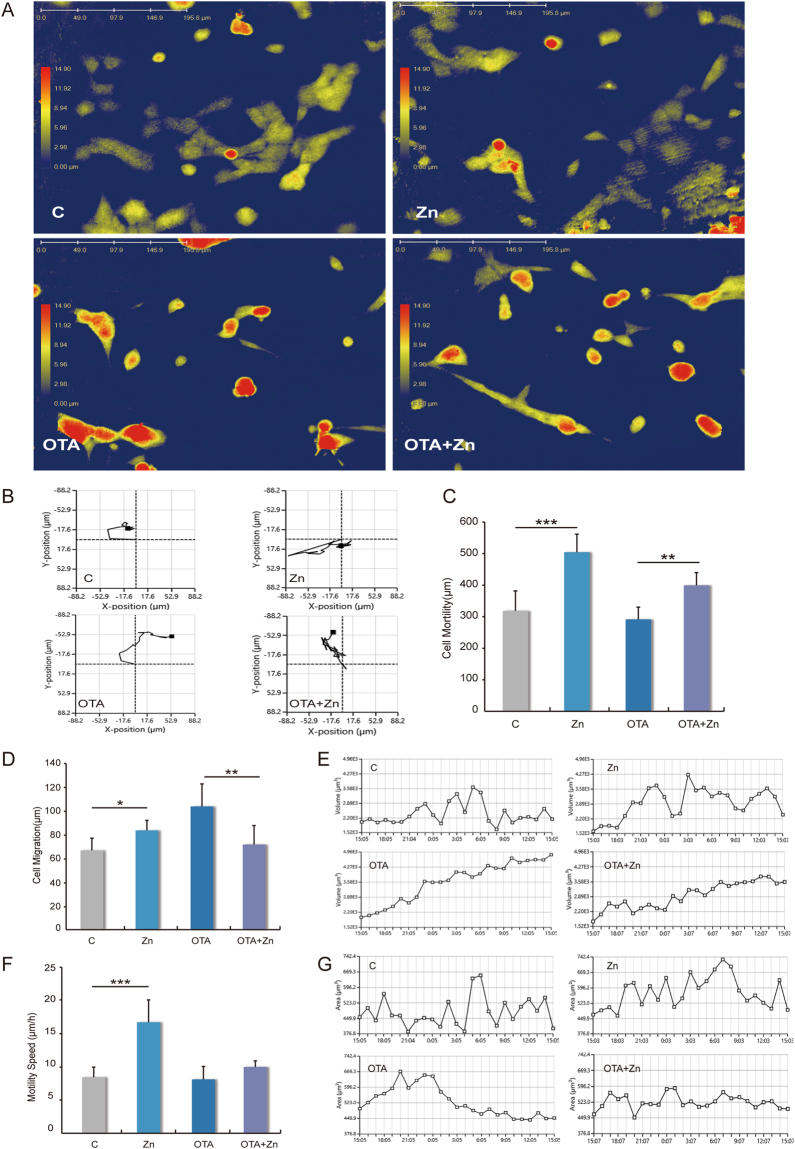
The motion performance of zinc supplementation in HEK293 cells in normal and toxic environments. (**A**) The holographic imaging records, based on interfering wave fronts from a laser of HEK-293 cells (2 × 10^5^), plated in culture media containing 10% FBS, 100 U/mL penicillin, 100 μg/mL streptomycin, 250 ng/mL amphotericin (**B**), 2 mM L-glutamine, and 1% MEM nonessential amino acids. Cells were treated with 50 μM ZnSO_4_ (group Zn), 25 μM OTA (group OTA), 50 μM ZnSO_4_ and 25 μM OTA (group OTA + Zn), or serum-free medium as a control (group C) for 24 h. The motion performance of (B) the motion distance, (**C**) the cell motility, (**D**) the cell migration, (**E**) the cell volume changes, (**F**) the motility speed and (**G**) the cell area changes were analyzed using the HoloStudio^TM^ software. In (**E**,**G**), the X axis represents the time variable. The values in each set are counted of twelve independent data points. The data were analyzed using one-way ANOVA. “*” indicates a significant difference compared with the control group p < 0.05, “**” indicates p < 0.01, “***” means p < 0.005.

To address the influence of zinc in cell morphology and motility, 3D holographic imaging records of an unfixed object in real time were used, based on interfering wave fronts from a laser (Fig. 1A). Dynamic changes in the cells were recorded as well (Video. 1). Compared to the control groups, zinc improved the motion distance (Fig. 1B). Quantitative analyses of the cell moving distance shows that in both normal and toxic cellular environments, 24 h 50 μM zinc supplementation improved cell motility by approximately 1.5 times in HEK293 cells (Fig. 1C). In addition, the cell migration and cellular displacement also showed that zinc benefits cell motility (Fig. 1D). While different cell lines grew with adherence, OTA could lead to cellular apoptosis^40^. The suspending death cells had increased motility, and this dynamic increase in cell motility was constitutionally distinguished with zinc in migration improvements. We observed that OTA could lead to the changing morphology of adherent cells, from a distended flattened appearance with stretching ability to a fully rounded shape with occasional surface blebs, suggesting a loss of cytoskeletal integrity or even autocytolysis (Fig. 1E). Zinc supplementation maintained an adherent morphology in the presence of 25 μM OTA for 24 h (Fig. 1E and G). It is noteworthy that zinc also can speed up HEK293 cells movement from 8 μm/h to 16 μm/h (Fig. 1F), but this was not significant in OTA-induced stress situation.

### Moderate zinc supplementation maintains cell viability and superoxide dismutase (SOD) activity

**Figure 2:**
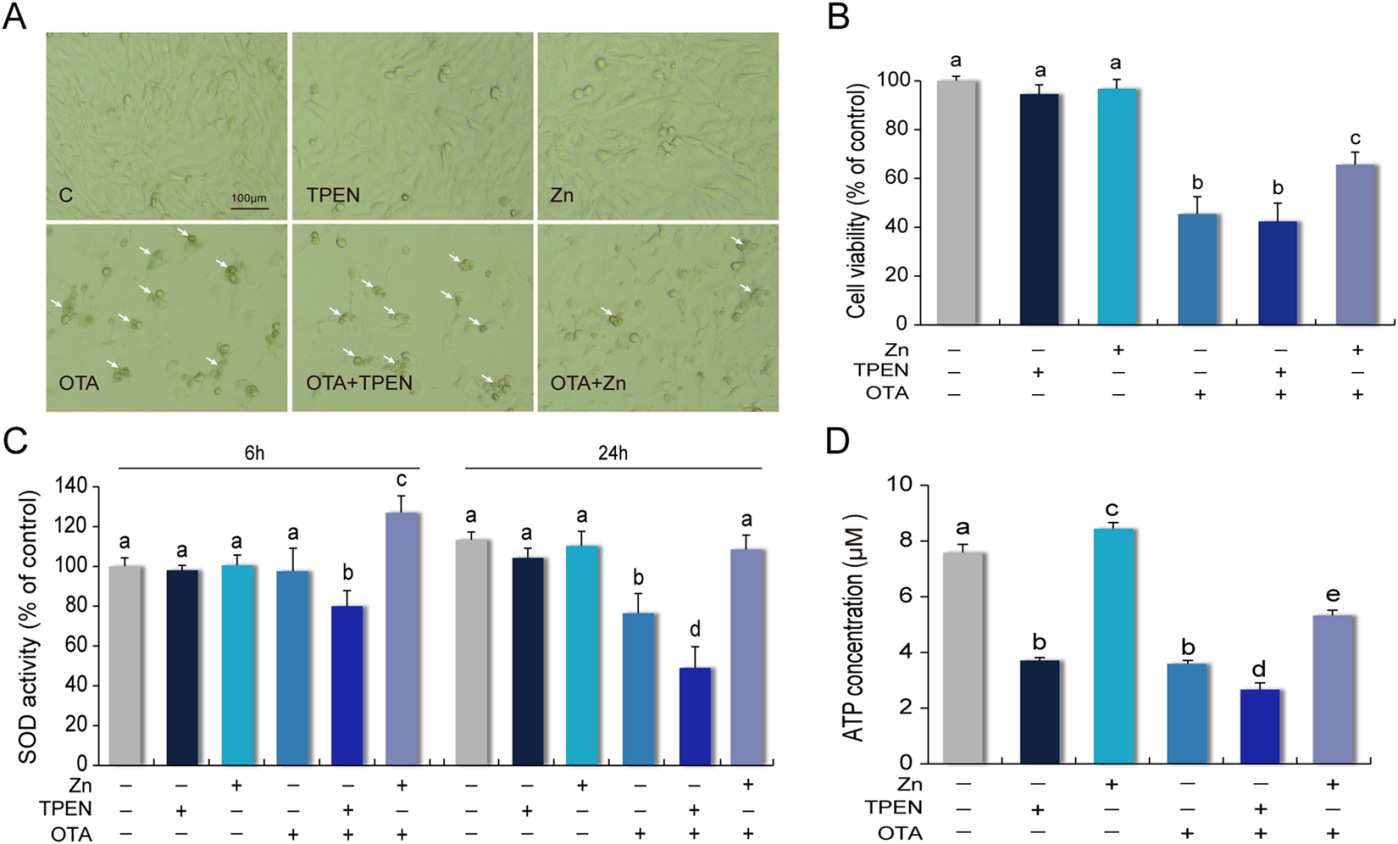
Zinc contributes to the restoration of cell viability, SOD and ATP activity in toxic environments. (**A**) The morphological observation of zinc in anti-OTA-induced adversity condition. (**B**) The cell viability was determined after 24 h using the CCK8 kit followed by absorbance detection at 450 nm. (**C**) The SOD activity of the treated cells in a 6-well plate were measured after 6 h and 24 h. (**D**) The ATP concentration of the treated cells were measured after 24 h, by using a microplate luminometer. All the data were expressed as a percentage of the control. The data are presented as the means ± SD of three independent experiments. The data were analyzed using one-way ANOVA. Different characters indicate significant differences between the compared groups (p < 0.05).

Of note, supraphysiological concentrations of TPEN are cytotoxic, but appropriate concentrations were suggested for an evaluation model that is sufficient to induce zinc deprivation, but not sacrifice cell viability^41,42^. OTA has a half maximal inhibitory concentration (IC50) of 25 μM and leads cellular DNA damage, cell cycle arrest and apoptosis. It is regarded as a toxic stress manifestation model^26,43^. Here, we confirmed this relationship among biologically effective doses for the performance. The apoptotic bodies were significantly decreased by zinc supplement in an OTA-induced toxic environment (Fig. 2A). The quantitative relationship with CCK-8 showed that 25 μM OTA reduced the cell viability to 48%. In addition, consistent with previous reports, 50 μM ZnSO_4_ had little effect on cell viability and morphological changes^44–48^. But notably, 50 μM zinc supplement in the OTA + Zn groups improved 20% of the cell viability compared with the OTA groups (Fig. 2B). This result showed that moderate zinc supplementation can maintain cell viability. In addition, the SOD activity was captured (Fig. 2C). Under an exposure time of OTA treatment for 6 h, the SOD activity was increased by zinc supplementation and decreased following zinc chelation. Over 24 h, this trend became clear. Chelated zinc in an OTA-induced toxic environment caused the SOD activity to drop to 58% of the control group.

### OTA-induced energy metabolism impairment was relieved by zinc but worsened with zinc chelation

**Figure 3:**
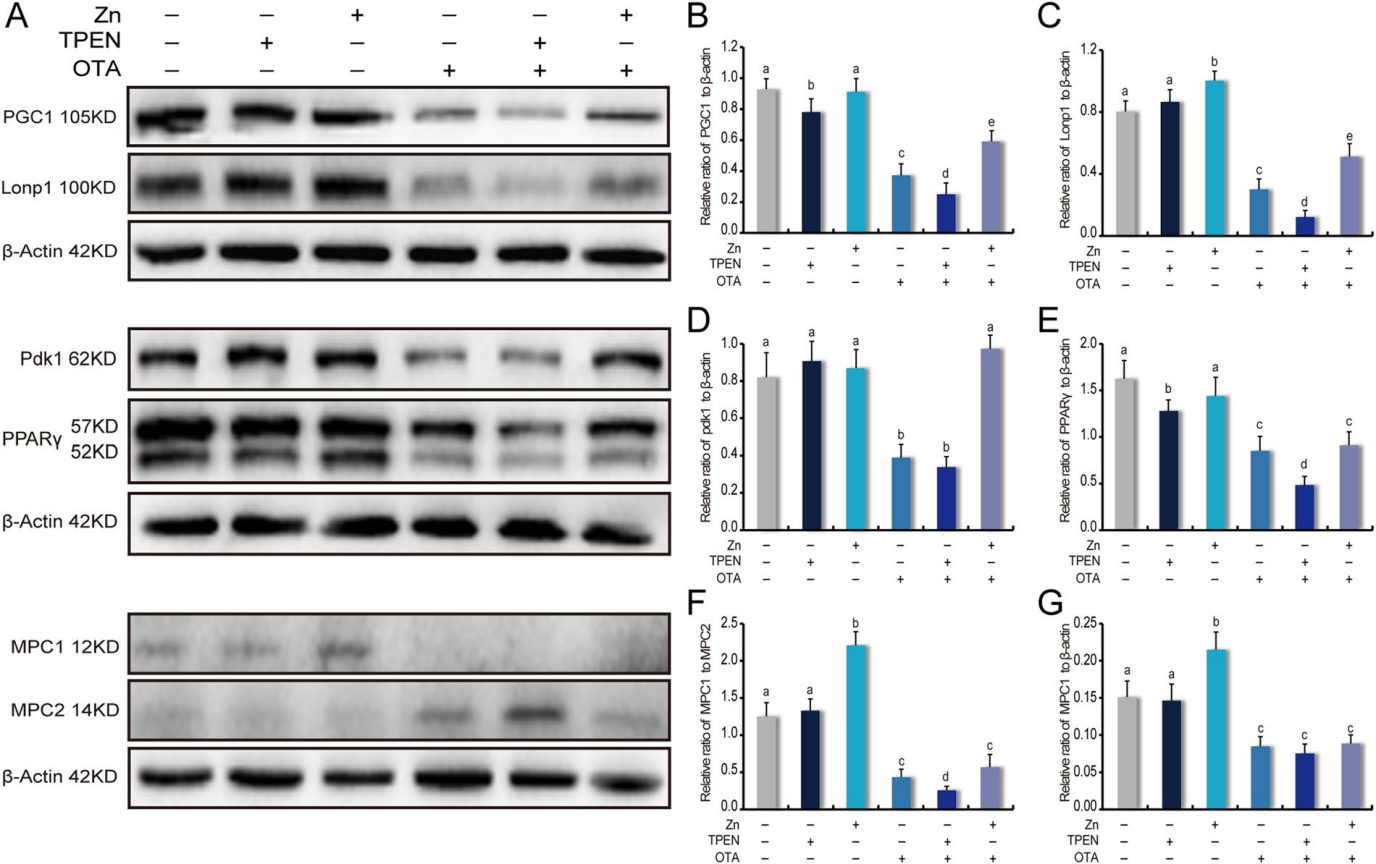
Zinc functions to restore impaired energetic metabolism pathways. (**A**) The expression levels of energy metabolism-associated proteins were detected by Western blotting. The expression levels of (**B**) PGC1α, (**C**) Lonp1, (**D**) PDK1 and (**E**) PPARγ were normalized to β-Actin expression. (**F**) The expression of the MPC1:MPC2 ratio. (**G**) The expression levels of MPC1 were normalized to the β-Actin expression levels. The values are expressed as the means ± SD of three independent experiments. The data were analyzed using one-way ANOVA. Different characters indicate significant differences between the compared groups (p < 0.05).

After exposure for 24 h, the ATP concentrations in both the OTA and TPEN groups were decreased by 50% (Fig. 2D). Zinc supplement increased to 30% of the ATP concentration relative to zinc chelated under OTA exposure conditions. Furthermore, a group of regulators involved with energy metabolism were investigated under the OTA-induced toxicant environment (Fig. 3A). PGC1α is a master regulator of mitochondrial biogenesis and regulates energy metabolism^49^. Lonp1 is an ATP-dependent serine protease in the mitochondrial matrix^50^. In adversity, relative to the OTA and zinc chelation groups, zinc promotes the up-regulation of PGC1α by 1.6 times and 2.2 times; the upregulation of Lonp1 by 1.3 times and 3.0 times (Fig. 3B and C). PDK1 and PPARγ are also essential in energy homeostasis and stress response^51,52^. Although zinc supplementation showed limited proactive roles in stimulating PDK1 activation in normal conditions, under the OTA-induced PDK1 inhibition, zinc supplementation kept the expression of PDK1 at a high level, almost 3 times more than in the zinc chelated groups (Fig. 3D). The expression of PPARγ was not sensitive to additional zinc supplementation, but zinc chelation by 5 μM TPEN downregulated PPARγ by more than 50% compared to the OTA treatment group (Fig. 3E).

### MPC complexes and pyruvate transport were regulated by zinc supplementation

MPC activation could obviously improve the TCA cycle and energy provision. MPC1 and MPC2 are required for formation of the functional MPC complex and effectual pyruvate transport. The MPC1:MPC2 ratio is an effective indicators used to evaluate the function of MPC complexes, beyond just assessing decreased intact MPC complexes and pyruvate transport^33^. Here, we observed that in the normal environment, zinc supplements could improve the MPC1:MPC2 ratio 1.8 times more than the control (Fig. 3F). This changes was dependent on the up-regulated expression of MPC1 (Fig. 3G). Zinc deficiency did not contribute to changes in pyruvate transport. However, in the OTA-induced toxic environment, the expression of MPC2 played a key role (Fig. 3F), which increased in OTA treatment and was strengthened in zinc deficiency, such the corresponding MPC1:MPC2 ratio decreased.

### Zinc remodeling of OXPHOS complexes occurs primarily around SDHB and NDUFB8 in toxic stress

**Figure 4:**
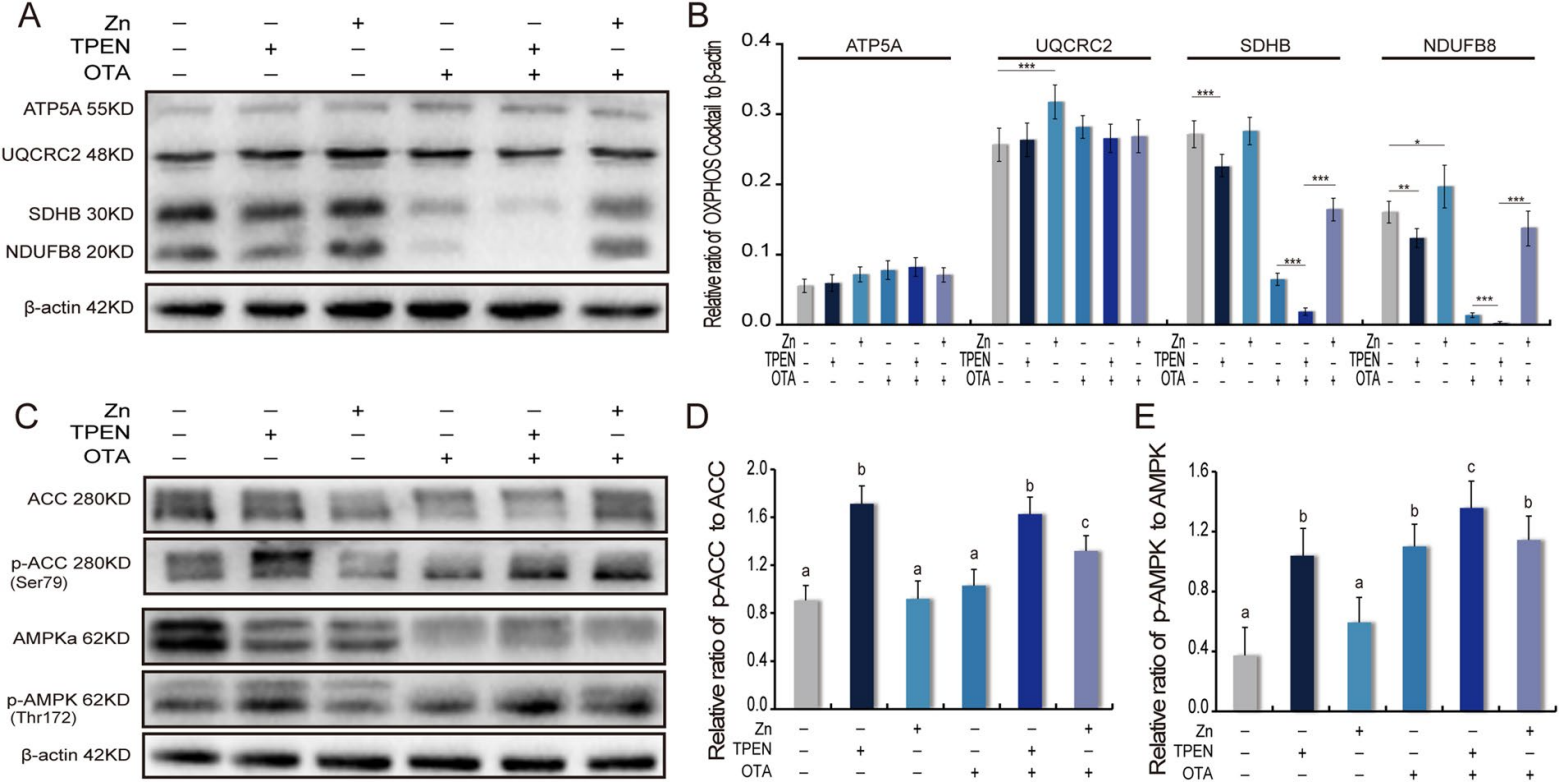
Zinc remodeling impaired OXPHOS complexes and is essential in stabilizing AMPK phosphorylation. (**A**) The expression of OXPHOS complex proteins was detected by Western blotting. (**B**) The relative quantity of OXPHOS complex proteins was normalized to the β-Actin expression. (**C**) The phosphorylation degree of AMPK in Thr172 and ACC in Ser79 was detected by Western blotting. (**D**) The phosphorylation degree of AMPK in Thr172 was normalized to AMPK expression. (**E**) The phosphorylation degree of ACC in Ser79 was normalized to ACC expression. The values are expressed as the means ± SD of three independent experiments. The data were analyzed using one-way ANOVA. “*” indicates a significant difference compared with the control group p < 0.05, “**” indicates p < 0.01, “***” means p < 0.005. Different characters indicate significant differences between the compared groups (p < 0.05).

Oxidative phosphorylation (OXPHOS) is an essential process for most ATP generation. Here, four members of the OXPHOS complexes, the alpha subunit of F-1-ATP synthase (ATP5A), the ubiquinol-cytochrome-c reductase core protein 2 (UQCRC2), the succinate dehydrogenase B (SDHB) and the NADH dehydrogenase (ubiquinone) I beta subcomplex 8 (NDUFB8) were investigated to show the function of zinc on OXPHOS (Fig. 4A). In our results, the expression of ATP5A was not sensitive to the addition of a zinc supplement or to zinc deficiency (Fig. 4B). UQCRC2 is a component of the ubiquinol-cytochrome c reductase complex and is associated with the mitochondrial respiratory complexes and ATP generation capacity^53^. Zinc supplementation led to a 1.3 times up-regulation of UQCRC2 compared to the control. In general, the expression of UQCRC2 was not sensitive to the concentration of zinc in either normal or toxic stress conditions (Fig. 4B). However, zinc played obvious and key roles in the SDHB and NDUFB8 of OXPHOS. In normal conditions, zinc supplementation led to a 1.2 times up-regulation of the expression of NDUFB8. To show the expression of SDHB and NDUFB8 in zinc deficiency, TPEN were treated and both regulators were shown to be down-regulated by 1.3 times (Fig. 4B). Of note, in the OTA-induced groups this trend was amplified. OTA induced a 3.5 times and 7.1 times down-regulation in the expression of SDHB and NDUFB8, respectively. Interestingly, chelated zinc by TPEN induced a further 4.2 times and 7.6 times down-regulation in the expression of SDHB and NDUFB8, respectively, compared to OTA alone. In contrary, zinc supplementation promoted SDHB and NDUFB8 expression to 2.4 times and 8.2 times the levels under OTA conditions (Fig. 4B). To our knowledge, this is the first study indicating an important role for zinc in SDHB and NDUFB8 regulation.

### Zinc is essential in stabilizing the homeostasis of the AMPK pathway

AMPK is activated by Thr172 phosphorylation^37^. As a substrate of AMPK, phosphorylation of ACC in ser79 was up-regulated 1.8 times and 1.6 times by TPEN in normal or toxic conditions (Fig. 4C and D). Accordingly, the Thr172 phosphorylation of AMPK was up-regulated 2.5 times and 1.3 times by TPEN in normal or toxic conditions, respectively (Fig. 4C and E).

## Discussion

OTA can inhibit the metabolism of energy by regulating the Krebs cycle, glycometabolism, arginine and proline metabolism, cysteine and methionine metabolism, and inhibiting the PPAR signaling pathway, resulting in metabolic disturbances^27,54^. The connection between zinc homeostasis and OTA toxicity in the intestinal epithelium has previously been reported^55^. OTA exposure can modulate the expression of zinc transporters ZnT and ZIP, especially ZnT1^55^, and reduce the zinc content in the cell total protein^56^. Besides, OTA-induced oxidative stress can regulate the expression of MTs^55^, which is an important ligand form of cellular zinc, and the zinc-MTs complex was an effective chaperone and donor for delivery and uptake of zinc by mitochondria^57^. Besides, the OTA treatment in zinc depleted cells reflects a mechanism that maintains intracellular zinc homeostasis and may lead to profound changes in zinc dependent energy metabolism impairment.

OXPHOS is a metabolic pathway in which cells use enzymes to oxidize nutrients, thereby releasing energy that is in turn used to reform ATP. The NADH dehydrogenase complex is essential in the oxidative phosphorylation process. Here, we first show that the restorative function of zinc is high relative to the expression of NDUFB8. NDUFB8 is mediated by the overexpression of zinc-associated mitochondrial superoxide dismutase^58^ and is considered to be an important regulator of the zinc-mediated restoration of mitochondrial homeostasis. UQCRC2 is including in oxidative phosphorylation and cellular energy metabolism promotion. Interestingly, UQCRC2 is known to be correlated with male fertility via spermatogenesis^59,60^. In sperm, it is generally accepted that zinc is an essential trace element for the maintenance of sperm functions, the progression of spermatogenesis and the regulation of sperm motility, TPEN treatment of sperm suppresses the rate and duration of their motility^61,62^. In the present study, the capacity of zinc in cell motility improvement was seen not only in sperm but also in cell motility regulation in general cell types.

Succinate dehydrogenase subunit B (SDHB) participates in both the citric acid cycle and the electron transport chain to promote energy metabolism^63^. SDHB loss-of-function mutations lead to mitochondrial enzyme SDH dysfunction, TGF-β signaling activation, and inflammation and are associated with tumor formation^64^. SDH-deficient tumors are believed to have a strong syndromic and hereditary basis and distinct natural history^65^. Currently, SDH-deficient renal carcinoma^66^, pituitary carcinoma^67^ and nasopharyngeal carcinoma^68^ are being discovered and classified. Here, given the strong regulation of zinc, we speculate that the hereditary nature of SDH-deficient tumors is relative to the long-term zinc deficiency of evolutionary history and that dietary zinc supplementation is one possible way to prevent or even treat SDH-deficient carcinomas.

Lonp1 also plays a key role in metabolic reprogramming by remodeling OXPHOS complexes and protecting against senescence^31^. Lonp1 is also a mitochondrial DNA binding protein that is important for mtDNA maintenance and is associated with the tricarboxylic acid cycle by degrading the oxidized aconitase^32^. To our knowledge, no previous researches has determined the relationship between zinc and Lonp1. Lonp1 can be down-regulated by OTA^69^, and both Lonp1 and zinc provide cytoprotection against oxidative stress^32^. Here, zinc supplementation has promoted Lonp1 expression in both normal and toxic environments. In addition, the dose-response relationship between zinc and Lonp1 provokes the cytoprotection of zinc in DNA methylation and epigenetic modifications^70^ could be achieved through Lonp1. Furthermore, Lonp1 deficiency induced insufficient energy supply, DNA instability, senescence or even apoptosis^31,32^ can be alleviated by zinc supplementation.

The mitochondrial import of pyruvate by the MPC is a key component of the mitochondrial pyruvate carrier which links cytosolic and mitochondrial intermediary metabolism^71,72^. MPC deficiency causes reduced contribution of glucose-derived pyruvate to the TCA cycle, diminishing TCA cycle intermediates, energy deficit and a perturbed balance of neurotransmitters^73^. Zinc is closely linked with neurotransmitters^74^ and has an effective role in preventing adverse outcomes of pregnancy and reducing the risk of reproduction disease in human and mammal^75,76^. In the present study, we therefore suspected that zinc-associated physiological protection could be involved with MPC, which activates the uptake of pyruvate, thus regulating the metabolic state necessary for embryonic development, neurotransmitter balance and post-natal survival^73^. Moreover, zinc deprivation tends to decrease mitochondrial pyruvate uptake and utilization, especially in adverse situations. The reduced MPC activity is an important aspect of cancer metabolism. Typically, cancer cells appear to select against MPC expression^33^, because altered MPC1/MPC2 expression or activity may result in significant metabolic disorders and contribute to an increase in aerobic glycolysis in cancer cells (a.k.a., the Warburg effect)^77^, so that increased MPC activity may decrease cancer proliferation^78^. Although the enhancement of cell motility could be relative to cancer proliferation, the zinc-induced increase in MPC activity seems to not agree with this point. This may be because cell motility and proliferation occur through different biological processes.

PDK1 is necessary for the complete and stable activation of protein kinase C (PKC), which is involved in the regulation of cell polarization, directional sensing, cell migration and particularly cell motility^79–81^. Faced with toxicity-induced stress, we showed that zinc supplementation promoted cellular anti-adversity, motility and PDK1 expression. Considering that PDK1 activation is essential in the central control of energy homeostasis and stress response through the phosphatidylinositol-3 kinase (PI3K) signaling pathway^51,82^, PDK1 was regarded to be a major site for zinc in restoring the impaired energetic metabolism.

Except for the activity index of normal cells or sperms, cell motility is also an important parameter used in the study of cancer metastasis^83^. Here, we confirmed that zinc can restore impaired energetic metabolism of cells under toxic stress, but this is a double-edged sword because zinc does not play active roles in acquired cancer therapy. This could be because zinc can upregulate the expression of the mammalian target of rapamycin (mTOR) kinase, which is a master regulator of protein synthesis that couples nutrient sensing to cell growth and cancer^84,85^. Zinc is also helpful to vessels and aids in the resistance to vascular diseases^86^, which is believed to be good news for tumor proliferation. Furthermore, the results of a phase III double-blind, placebo-controlled trial did not show that zinc sulfate could prevent taste alterations in cancer patients who were undergoing radiotherapy of the oral pharynx^87^. Regardless, dietary deficiencies in zinc absolutely contribute to single- and double-stranded DNA breaks and oxidative modifications to DNA that increase the risk for cancer development^88^.

The energy sensor AMPK acts as a regulator of energy balance at both the cellular and the whole-body levels by stimulating ATP-generating pathways and inhibiting ATP-consuming anabolic pathways^89^. The overall metabolic consequence of AMPK activity is the maintenance of energy levels under ATP-limiting conditions^90^. In this regard, AMPK is highly sensitive to AMP, as any increase in the AMP/ATP ratio due to a decrease in the cellular energy state, stimulates AMPK activity^91^. Activated AMPK stimulates glucose uptake and lipid oxidation in peripheral tissues^92^ and plays an important role in maintaining the energy balance of eukaryotes, where AMPK activity can be regulated by a wide array of factors^93^. A decrease in cellular ATP and a concomitant increase in AMP levels triggers the phosphorylation-dependent activation of AMPK at Thr-172^94^.

PGC1α is a transcriptional coactivator, the master regulator of mitochondrial biogenesis that regulates energy metabolism and linking environmental stimulus to adaptive thermogenesis^49,95^. Although it is not physiologically stimulated by zinc under normal conditions, in the conditions of oxidative stress or adverse environments, zinc makes a considerable degree of callback in the expression of PGC1α. Considering that PGC1α has been proposed to be responsible for muscle fiber type determination and β-aminoisobutyric acid secretion by exercising muscles^96,97^, it is not surprising that zinc supplementation not only reinforced energetic metabolism but also relieved some of the pressure for the oxidative stress.

**Figure 5:**
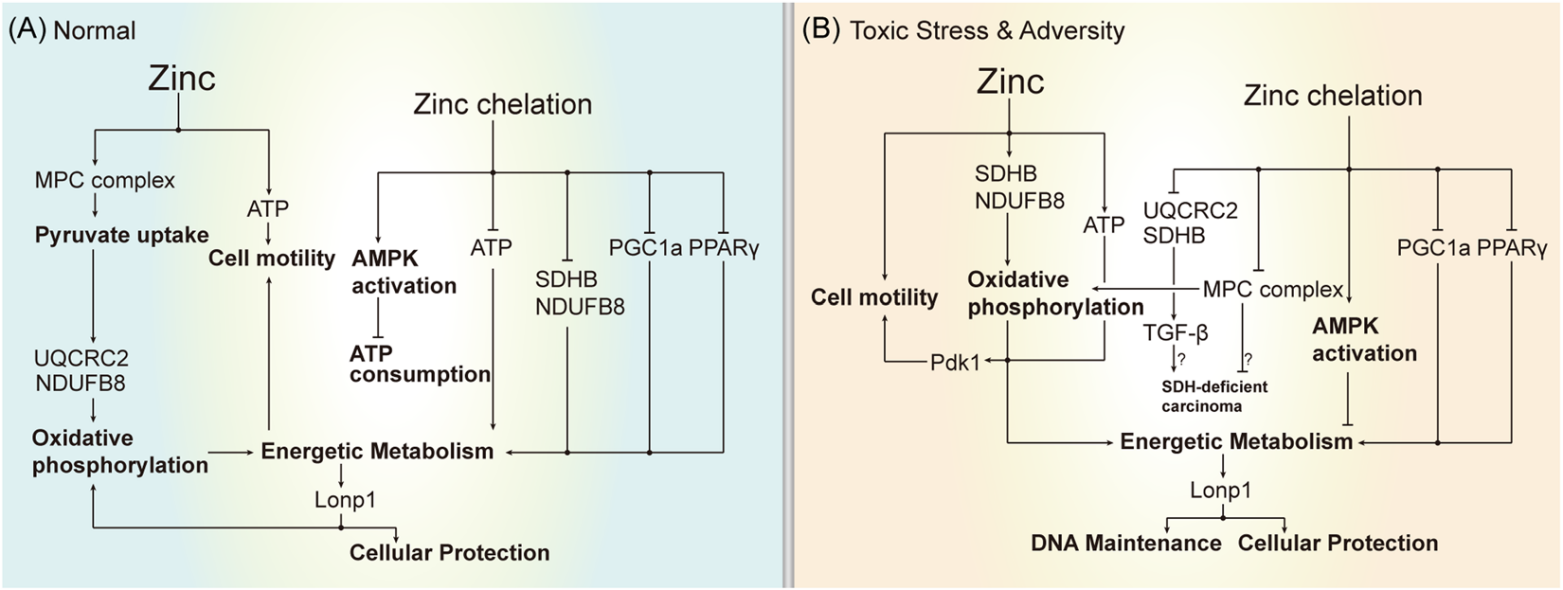
Model for how zinc participates in energy metabolism pathways in both normal and OTA-induced adversity conditions. (**A**) In the normal condition, zinc is conducive to mitochondrial pyruvate transport, UQCRC2 and NDUFB8-related oxidative phosphorylation, cell motility and Lonp1-related energy metabolism regulation. Zinc chelation suppress ATP contents, AMPK activation, oxidative phosphorylation, PGC1α-, PPARγ-mediated energy metabolism, thus having adverse effects on energetic metabolism. (**B**) In toxin-induced adversity, zinc is helpful to restore the impaired energetic metabolism by the expression of Lonp1, PGC1α, PDK1, NDUFB8, UQCRC2, and SDHB. In addition to cell motility, protection and DNA maintenance could also be reinforced. In addition, zinc deficiency could be related to the development of SDH-deficient carcinoma through the regulator SDHB and MPC complexes^64,65,77^.

Zinc participates in the cellular energetic metabolism enhancement pathways of HEK293 cells in both normal and toxin-induced conditions. Its roles are summarized in Fig. 5. First, zinc is conducive to mitochondrial pyruvate transport. Second, especially since UQCRC2 and SDHB in oxidative phosphorylation are sensitive to zinc in both normal and toxic environments, it directly promotes energetic metabolism. Third, the promotion of energetic metabolism and PDK1 could be an important aspect of zinc-mediated cell motility improvement^8^. Fourth, in the opposite case, zinc chelation suppresses ATP content, pyruvate transport, AMPK-, PGC1α- and PPARγ-mediated energy metabolism and homeostasis, thus aggravating the impaired energetic metabolism. Our results suggest that zinc can improve the cell motility and energy metabolism of normal cells *in vitro* and restore the expression of Lonp1, PGC1α, PDK1, UQCRC2 and SDHB in toxin impaired energetic metabolism. Our results also suggest that the hereditary characteristics of SDH-deficient tumors could be relative to long-term zinc deficiency, and dietary zinc supplementation is a possible way to prevent SDH-deficient carcinoma.

## Electronic supplementary material

Video 1A

Video 1B

Video 1C

Video 1D
